# The influence of gender on the epidemiology of and outcome from severe sepsis

**DOI:** 10.1186/cc12570

**Published:** 2013-03-18

**Authors:** Yasser Sakr, Cristina Elia, Luciana Mascia, Bruno Barberis, Silvano Cardellino, Sergio Livigni, Gilberto Fiore, Claudia Filippini, Vito Marco Ranieri

**Affiliations:** 1 Department of Anesthesiology and Intensive Care, Friedrich-Schiller-University, Erlanger Allee 103, 07743 Jena, Germany; 2 Department of Anesthesiology and Intensive Care, San Giovanni Battista-Molinette Hospital, University of Turin, corso Dogliotti 14, 10126 Turin, Italy; 3 Department of Anesthesiology and Intensive Care, Ospedale degli Infermi, via Rivalta 29, 10128 Rivoli (TO), Italy; 4 Department of Anesthesiology and Intensive Care, Ospedale Cardinal Massaia, corso Dante 202, 14100 Asti, Italy; 5 Department of Anesthesiology and Intensive Care, Ospedale Giovanni Bosco, piazza Donatore di sangue n° 3, 10154 Turin, Italy; 6 Department of Anesthesiology and Intensive Care, Ospedale Santa Croce, Piazza A. Ferdinando n° 3, 10024 Moncalieri (TO), Italy

## Abstract

**Introduction:**

The impact of gender on outcome in critically ill patients is unclear. We investigated the influence of gender on the epidemiology of severe sepsis and associated morbidity and mortality in a large cohort of ICU patients in the region of Piedmont in Italy.

**Methods:**

This was a *post-hoc *analysis of data from a prospective, multicenter, observational study in which all patients admitted to one of 24 participating medical and/or surgical ICUs between 3 April 2006 and 29 September 2006 were included.

**Results:**

Of the 3,902 patients included in the study, 63.5% were male. Female patients were significantly older than male patients (66 ± 16 years vs. 63 ± 16 years, *P *< 0.001). Female patients were less likely to have severe sepsis and septic shock on admission to the ICU and to develop these syndromes during the ICU stay. ICU mortality was similar in men and women in the whole cohort (20.1% vs. 19.8%, *P *= 0.834), but in patients with severe sepsis was significantly greater in women than in men (63.5% vs. 46.4%, *P *= 0.007). In multivariate logistic regression analysis with ICU outcome as the dependent variable, female gender was independently associated with a higher risk of ICU death in patients with severe sepsis (odds ratio = 2.33, 95% confidence interval = 1.23 to 4.39, *P *= 0.009) but not in the whole cohort (odds ratio = 1.07, 95% confidence interval = 0.87 to 1.34).

**Conclusion:**

In this large regional Italian cohort of ICU patients, there were more male than female admissions. The prevalence of severe sepsis was lower in women than in men, but female gender was independently associated with a higher risk of death in the ICU for patients with severe sepsis.

## Introduction

During the past decade, several clinical and epidemiological studies have investigated the impact of gender on outcome in various clinical settings, yielding conflicting results [1-10]. Sexual dimorphism in the immune response to noxious agents has been correlated to differences in sex steroid hormone concentrations that ultimately determine the effect of gender on outcome [11-13]. Females have been observed to have more prominent hormonal and cell-mediated immune responses compared with males. Schröder and colleagues demonstrated that male patients with sepsis had testosterone levels that were consistently lower than the normal range and that postmenopausal female patients had higher estradiol levels than expected [14]. These differences in hormonal secretion may play a key role in the improved survival of critically ill women. Moreover, dysregulated proinflammatory and anti-inflammatory responses related to sexual immunomodulation of the cytokine network are thought to be responsible for differences in susceptibility to sepsis and subsequent multiorgan failure, which correlate with sex-based mortality rates [12,15]. A recent French study, however, found that mortality was higher among female ICU patients developing nosocomial infections than among male patients [10]. A higher risk of in-hospital death was also found for younger women undergoing coronary artery bypass surgery [4] and for female trauma patients who acquired pneumonia during the ICU stay [3].

We conducted this *post-hoc *analysis to investigate the influence of gender on the epidemiology of severe sepsis in a large cohort of ICU patients in the region of Piedmont in Italy and its possible impact on morbidity and mortality in these patients.

## Materials and methods

All adult patients (> 18 years old) admitted to the 24 Italian ICUs participating in the Piedmont Intensive Care Unit Network were included in this prospective multicenter observational study conducted between 3 April 2006 and 29 September 2006 [16,17]. These ICUs represent 75% of the ICUs in the region of Piedmont; in particular, peripheral and central hospitals of the provinces of Torino, Cuneo, Asti and Alessandria. Recruitment for participation was by open invitation and was voluntary, with no financial incentive. The study was approved by the institutional review board of the coordinating center (San Giovanni Battista-Molinette Hospital, University of Turin, Italy) and adopted by the participating centers (Additional file 1). Informed consent was not required because of the observational nature of the study.

Data collection was performed using database-oriented software. For all variables collected, precise definitions were provided in the relevant part of the software. In each ICU, a trained physician was responsible for data collection and entry. Central support was provided by the department of anesthesiology and intensive care at the University of Turin (coordinating center). Validity checks were made concurrent with data entry in the electronic case record form, including plausibility checks for each variable and between variables. Data were further reviewed by the coordinating center, and any doubts clarified with the corresponding ICU.

For all patients, the following data were recorded on admission to the ICU: demographics (age, sex), admission diagnoses, admission category (medical, scheduled surgery, emergency surgery, or trauma) and origin (emergency, surgical or medical ward or another ICU from the same hospital, or transfer from another hospital), comorbidities, surgical status, reason for admission, and the components of the Simplified Acute Physiology Score (SAPS) II [18]. Daily data collection included the presence of systemic inflammatory response syndrome. Patients with > 2 systemic inflammatory response syndrome criteria were additionally screened for the presence of infection, and the parameters of organ dysfunction/failure as assessed by the Sequential Organ Failure Assessment (SOFA) score [19] were recorded daily thereafter by the attending physician. Patients were followed up from the first day of admission until death or ICU discharge. Only the first admission to the ICU was considered.

### Definitions

Sepsis syndromes were diagnosed according to the criteria proposed by the American College of Chest Physicians/Society of Critical Care Medicine Consensus Conference [20]. ICU-acquired sepsis was defined as sepsis identified > 48 hours after ICU admission and non-ICU-acquired sepsis as sepsis occurring within 48 hours of ICU admission. Surgical admissions were defined as patients who had undergone surgery within 2 weeks preceding admission. Emergency surgery was defined as a nonscheduled operation within 24 hours of the onset of symptoms or injury. Comorbidities included the presence of insulin-dependent diabetes mellitus (need for daily injection of insulin prior to ICU admission), chronic obstructive pulmonary disease, heart failure class III or IV according to the New York Heart Association definitions, and chronic renal failure (need for chronic renal support or history of chronic renal insufficiency). Patients were also classified by the admitting physician according to whether they were admitted to the ICU for only short-term monitoring, had an expected ICU length of stay < 24 hours, or were admitted for intensive care treatment with an expected ICU length of stay > 24 hours.

We also examined the epidemiology of severe septic syndromes in two *a priori *defined age subgroups of patients (≤ 50 years and > 50 years), assuming that a 50-year cutoff value represented a reasonable physiological limit between premenopausal and postmenopausal periods for women.

### Outcome parameters

The primary outcome parameter was death in the ICU. Secondary outcome parameters were the development of sepsis syndromes in the ICU and the ICU length of stay.

### Statistical analysis

Data were analyzed using SPSS 17.0 for Windows (SPSS Inc., Chicago, IL, USA). Discrete variables are expressed as counts (percentage) and continuous variables as means ± standard deviation or median and interquartile range unless stated otherwise. Categorical data were compared using the chi-square test with Yates' correction, by Fisher's exact test or by the Cochran-Armitage trend test, as appropriate. A Kolmogorov-Smirnov test was used to verify the normality of distribution of continuous variables. Continuous variables conforming to a normal distribution were compared using analysis of variance and Student's *t *test; otherwise the Kruskal-Wallis and Mann-Whitney U tests were applied. A Bonferroni correction was done for multiple comparisons. Kaplan-Meier survival curves stratified according to gender were plotted over the 28 days following admission to the ICU and were compared using a log-rank test.

To investigate the impact of gender on ICU mortality adjusting for differences in baseline characteristics and severity of illness, we performed a logistic regression analysis with ICU mortality as the dependent factor in the overall population. Variables included in this analysis were age, comorbid diseases, SAPS II on admission, the referring facility, the type of admission to the ICU, the presence of sepsis syndromes and the time of acquisition of sepsis. Collinearity between the variables was excluded prior to modeling. A Hosmer and Lemeshow goodness-of-fit test was performed, and odds ratios (ORs) with 95% confidence intervals (CIs) were computed. We adjusted for the center effect in the final model by introducing this as a covariate, with the center that included the largest number of patients as the reference category. A similar model was constructed in patients with severe sepsis. The source of infection, SOFA scores, and the time of acquisition of sepsis were also considered in this model. In the whole cohort, the multivariate analysis included all covariates. In patients with severe sepsis, however, covariates were included if *P *< 0.2 in a univariate logistic regression analysis in order to reduce the number of covariates in the model because of the relatively small number of patients in this subgroup.

All statistics were two-tailed and *P *< 0.05 was considered statistically significant.

## Results

### Characteristics of the study cohort

During the study period 3,902 patients were admitted to the participating centers, of whom 2,479 (63.5%) were male. The characteristics of the study group are shown in Table 1[16,17]. Female patients were significantly older than male patients (66 ± 16 years vs. 63.4 ± 15.6 years, *P *< 0.001). There were no significant differences between men and women in comorbidities, SAPS II, type and reason of admission, or referring facility. More of the patients admitted with trauma were male (15.8% vs. 8.6%, *P *< 0.001).

**Table 1:** Characteristics of the study cohort on admission to the ICU stratified according to gender

	All patients	Male	Female	*P *value
*n*	3,902	2,479	1,423	
Age (years)	64.3 ± 15.7	63.4 ± 15.6	66.0 ± 16	< 0.001
SAPS II	37.2 ± 17.7	37.2 ± 18	37.5 ± 17.5	0.326
Comorbidities				
Diabetes mellitus	586 (15)	361 (14.6)	225 (15.8)	0.293
Renal failure (without dialysis)	269 (6.9)	172 (6.9)	97 (6.8)	0.885
Renal failure (with dialysis)	97 (2.5)	55 (2.2)	42 (3)	0.157
Hematological cancer	65 (1.7)	44 (1.8)	21 (1.5)	0.518
Chronic heart failure (NYHA III to IV)	303 (7.8)	182 (7.3)	121 (8.5)	0.192
COPD	280 (7.2)	192 (7.7)	88 (6.2)	0.069
Type of admission				0.279
Elective surgery	1,515 (38.8)	967 (39)	548 (38.5)	
Emergency surgery	979 (25.1)	602 (24.3)	377 (26.5)	
Medical admission	1,408 (36.1)	910 (36.7)	498 (35)	
Reason for admission				0.179
Only monitoring	1,793 (46)	1,119 (45.1)	674 (47.4)	
Intensive care	2,109 (54)	1,360 (54.9)	749 (52.6)	
Trauma	514 (13.2)	392 (15.8)	122 (8.6)	< 0.001
Referring facility				0.087
Other hospital	445 (11.4)	287 (11.6)	158 (11.1)	
Surgical ward	1,789 (45.8)	1,106 (44.6)	683 (48)	
Emergency department	937 (24.0)	629 (25.4)	308 (21.6)	
Medical ward	625 (16.0)	389 (15.7)	236 (16.6)	
Other ICU	106 (2.7)	68 (2.7)	38 (2.7)	
ICU mortality	780 (20)	498 (20.1)	282 (19.8)	0.838
ICU length of stay (days)	3 (1 to 9)	3 (1 to 10)	3 (1 to 7)	0.154

Data presented as mean ± standard deviation, *n *(%) or median (interquartile range). COPD, chronic obstructive pulmonary disease; NYHA, New York Heart Association classification; SAPS, Simplified Acute Physiology Score.

### Impact of gender on the epidemiology of severe sepsis

The frequency of severe sepsis, including septic shock, during the ICU stay was lower in women than in men (6.0% vs. 8.9%, *P *= 0.001) irrespective of the age group, mainly because of the lower occurrence of these syndromes in women within 48 hours of admission to the ICU (2.3% vs. 4%, *P *= 0.005) (Table 2). The prevalence of septic shock was extremely low in female patients aged ≤ 50 years (0.9%). Among 305 patients who had severe sepsis or septic shock during the ICU stay, 220 (72.1%) were male and only 85 (27.9%) were female. Female patients with severe sepsis were older than male patients (67.7 ± 14.3 years vs. 63.1 ± 15 years, *P *= 0.004), but other baseline characteristics, the source of infection, and SOFA scores were similar irrespective of gender (Tables 3 and 4).

**Table 2:** Frequency of severe sepsis according to gender in the whole population and stratified by age

	All patients (*n *= 3,902)		Patients aged ≤ 50 years (*n *= 703)		Patients aged > 50 years (*n *= 3,199)	
	
	Male	Female	Male	Female	Male	Female
*N*	2,479	1,423	474	229	2,005	1,194
On admission to the ICU						
Severe sepsis and septic shock	119 (4.8)	51 (3.6)*	17 (3.6)	4 (1.7)*	102 (5.1)	47 (3.9)*
Septic shock	72 (2.9)	28 (2)*	12 (2.5)	0 (0.0)^†^	60 (3)	28 (2.3)*
Any time during the ICU stay						
Severe sepsis and septic shock	220 (8.9)	85 (6)^‡^	47 (9.9)	10 (4.4)^†^	173 (8.6)	75 (6.3)^†^
Septic shock	103 (4.2)	42 (3)*	23 (4.9)	2 (0.9)^‡^	80 (4)	40 (3.4)*
Severe sepsis within 48 hours of admission	99 (4)	33 (2.3)^‡^	30 (6.3)	6 (2.6)*	69 (3.4)	27 (2.3)*
ICU-acquired severe sepsis (> 48 hours)	121 (4.9)	52 (3.7)*	17 (3.6)	4 (1.7)*	104 (5.2)	48 (4)*

*P = 0.1 to 0.05, ^†^*P *= 0.05 to 0.01, ^‡^*P *< 0.01 compared with males.

**Table 3:** Characteristics of patients with severe sepsis stratified according to gender

	All patients	Male	Female	*P *value
*n*	305	220 (72.1)	85 (27.9)	
Age (years)	64.4 ± 14.9	63.1 ± 15.	67.7 ± 14.3	0.004
SAPS II	55.2 ± 17.8	55.3 ± 17.5	55 ± 18.8	0.972
Comorbidities				
Diabetes mellitus	52 (17)	34 (15.5)	18 (21.2)	0.234
Renal failure (without dialysis)	34 (11.1)	25 (11.4)	9 (10.6)	0.847
Renal failure (with dialysis)	22 (7.2)	15 (6.8)	7 (8.2)	0.668
Hematological cancer	10 (3.3)	6 (2.7)	4 (4.7)	0.384
Chronic heart failure (NYHA III to IV)	25 (8.2)	17 (7.7)	8 (9.4)	0.631
COPD	21 (6.9)	18 (8.2)	3 (3.5)	0.150
Type of admission				0.486
Elective surgery	25 (8.2)	17 (7.7)	8 (9.4)	
Emergency surgery	109 (35.7)	75 (34.1)	34 (40)	
Medical admission	171 (56.1)	128 (58.2)	43 (50.6)	
Reason for admission				0.211
Only monitoring	20 (6.6)	12 (5.5)	8 (9.4)	
Intensive care	285 (93.4)	208 (94.5)	77 (90.6)	
Trauma	32 (10.5)	27 (12.3)	5 (5.9)	0.103
Referring facility				0.532
Other hospital	53 (17.4)	42 (19.1)	11 (12.9)	
Surgical ward	83 (27.2)	57 (25.9)	26 (30.6)	
Emergency department	96 (31.5)	72 (32.7)	24 (28.2)	
Medical ward	57 (18.7)	38 (17.3)	19 (22.4)	
Other ICU	16 (5.2)	11 (5)	5 (5.9)	
ICU mortality	156 (51.1)	102 (46.4)	54 (63.5)	0.007
ICU length of stay (days)	13 (6 to 26)	13 (7 to 26)	9 (5 to 25)	0.115

Data presented as mean ± standard deviation, *n *(%) or median (interquartile range). COPD, chronic obstructive pulmonary disease; NYHA, New York Heart Association classification; SAPS, Simplified Acute Physiology Score.

**Table 4:** Characteristics of infections in patients with severe sepsis stratified according to gender

	All patients	Male	Female	*P *value
*n*	305	220 (72.1)	85 (27.9)	
Site of infection				
Pulmonary	183 (60)	138 (62.7)	45 (52.9)	0.118
Abdominal	112 (36.7)	77 (35)	35 (41.2)	0.316
Catheter-related	7 (2.3)	5 (2.3)	2 (2.4)	0.967
Renal	18 (5.9)	13 (5.9)	5 (5.9)	0.993
Central nervous system	9 (3)	5 (2.3)	4 (4.7)	0.260
Bone	4 (1.3)	4 (1.8)	0	0.211
Soft tissue	15 (4.9)	12 (5.5)	3 (3.5)	0.486
Unknown	38 (12.5)	22 (10)	16 (18.8)	0.036
Other	80 (26.2)	53 (24.1)	27 (31.8)	0.172
Initial SOFA subscores				
SOFA respiratory	3 (2 to 3)	3 (2 to 3)	2 (2 to 3)	0.312
SOFA hepatologic	0 (0 to 2)	0 (0 to 2)	0 (0 to 1)	0.439
SOFA hematologic	1 (0 to 2)	1 (0 to 2)	1 (0 to 2)	0.803
SOFA renal	1 (0 to 2)	1 (0 to 2)	1 (0 to 2)	0.254
SOFA neurologic	2 (1 to 3)	2 (1 to 3)	2 (1 to 3)	0.729
SOFA cardiovascular	4 (2 to 4)	4 (2 to 4)	3 (1.5 to 4)	0.282
SOFA scores during sepsis				
Initial SOFA score	9.6 ± 3.6	9.8 ± 3.7	9.1 ± 3.3	0.190
SOFA mean	9.4 ± 3.6	9.6 ± 3.7	8.9 ± 3.5	0.197
SOFA maximum	9.7 ± 3.6	9.9 ± 3.7	9.1 ± 3.3	0.151

Data presented as *n *(%) or median (interquartile range). SOFA, Sequential Organ Failure Assessment.

### Impact of gender on outcome

**Figure 1:**
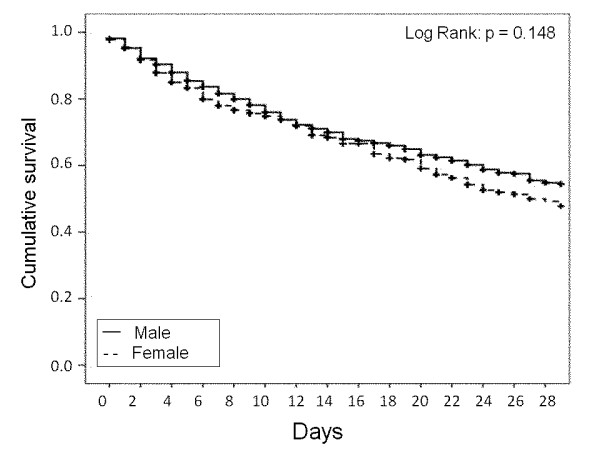
**Kaplan-Meier survival curves representing 28-day survival according to gender in the whole cohort**.

The overall ICU mortality rate was 20% and did not differ significantly between male and female patients (20.1% vs. 19.8%, *P *= 0.838). Twenty-eight-day survival rates, censored at ICU discharge, were similar in male and female patients (log-rank *P *= 0.148, Figure 1). The median ICU length of stay in the whole cohort was 3 (1 to 9) days (survivors vs. nonsurvivors, 18 (9.5 to 30) vs. 9 (4 to 21), *P *< 0.001) and was similar in men and women (Table 1). In a multivariate logistic regression analysis with ICU mortality as the dependent variable, female gender was not independently associated with an increased risk of death in the ICU (OR = 1.07, 95% CI = 0.87 to 1.34) (full results of the regression analysis are shown in Additional file 2).

**Figure 2:**
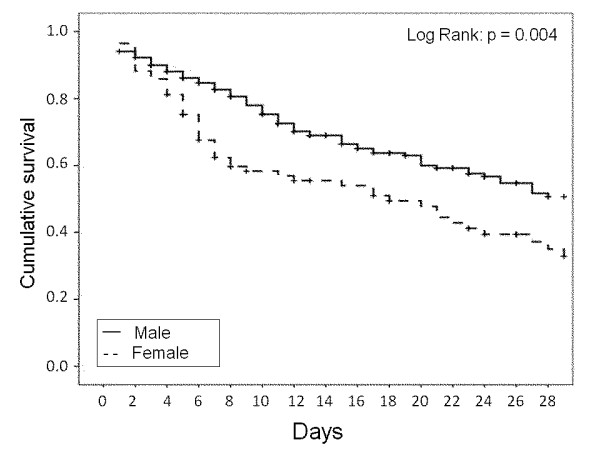
**Kaplan-Meier survival curves representing 28-day survival according to gender in patients with severe sepsis**.

In patients with severe sepsis, ICU mortality was 51.1% and was higher in women than in men (63.5% vs. 46.4%, *P *= 0.007). A Kaplan-Meier analysis showed reduced 28-day survival in female compared with male patients with severe sepsis (log-rank *P *= 0.004, Figure 2). However, the ICU length of stay was similar in men and women (Table 3). In a multivariate logistic regression analysis in patients with severe sepsis with ICU outcome as the dependent variable, age (OR = 1.03, 95% CI = 1.01 to 1.05, *P *= 0.02), female gender (OR = 2.33, 95% CI = 1.23 to 4.39, *P *= 0.009) and SAPS II (OR = 1.03, 95% CI = 1.01 to 1.05, *P *= 0.001) were independently associated with a higher risk of ICU death. Other factors associated with a higher risk of death in this population were SOFA respiratory and SOFA cardiovascular subscores, referral from a surgical ward, an emergency department or another ICU, and an abdominal site of infection (Table 5).

**Table 5:** Logistic regression analysis with ICU mortality as the dependent variable in patients with severe sepsis

	Univariate		Multivariate^a^	
	
	OR (95% CI)	*P *value	OR (95% CI)	*P *value
Age (per year)	1.03 (1.02 to 1.05)	< 0.001	1.03 (1 to 1.059)	0.016
Sex (female)	2.01 (1.20 to 3.37)	0.008	2.23 (1.17 to 4.24)	0.014
Comorbidities				
Diabetes mellitus	0.79 (0.43 to 1.45)	0.465		
Renal failure (with dialysis)	0.21 (0.07 to 0.64)	0.006	2.9 (0.68 to 12.45)	0.151
Renal failure (without dialysis)	1.05 (0.52 to 2.15)	0.887		
Heart failure (NYHA III to IV)	0.96 (0.42 to 2.19)	0.929		
COPD	0.50 (0.19 to 1.28)	0.147	1.75 80.58 to 5.32)	0.319
SAPS II (per point)	1.04 (1.03 to 1.06)	< 0.001	1.03 (1.01 to 1.05)	0.002
Type of admission				
Elective surgery	R	NA	R	NA
Emergency surgery	1.56 (0.64 to 3.84)	0.330	1.09 (0.38 to 3.5)	0.891
Medical admission	2.28 (0.95 to 5.43)	0.064	1.7 (0.5 to 5.76)	0.392
Initial SOFA subscores				
SOFA respiratory	1.53 (1.21 to 1.92)	< 0.001	1.65 (1.24 to 2.2)	0.001
SOFA hepatologic	1.18 (0.96 to 1.45)	0.110	1.25 (0.94 to 1.66)	0.126
SOFA hematologic	1.32 (1.08 to 1.60)	0.006	1.28 (0.99 to 1.65)	0.059
SOFA renal	1.29 (1.07 to 1.54)	0.006	0.92 (0.71 to 1.19)	0.535
SOFA neurologic	1.097 (0.94 to 1.29)	0.257		
SOFA cardiovascular	1.272 (1.09 to 1.49)	0.003	1.24 (1.02 to 1.51)	0.034
Referring facility				
Other hospital	R	NA	R	NA
Surgical ward	0.2 (0.09 to 0.45)	< 0.001	5.17 (1.2 to 22.29)	0.027
Emergency department	0.93 (0.28 to 3.1)	0.913	3.53 (1.52 to 8.21)	0.003
Medical ward	0.52 (0.26 to 1.05)	0.068	1.43 (0.52 to 3.93)	0.492
Other ICU	0.31 (0.15 to 0.63)	0.001	4.87 (1.85 to 12.85)	0.001
Source of infection				
Pulmonary	0.926 (0.586 to 1.465)	0.743		
Abdominal	0.542 (0.338 to 0.871)	0.011	2.51 (1.15 to 5.44)	0.02
Renal	1.697 (0.640 to 4.501)	0.288		
Central nervous system	0.833 (0.219 to 3.164)	0.789		
Bone	3.185 (0.328 to 30.965)	0.318		
Soft tissue	1.208 (0.427 to 3.417)	0.722		

Logistic regression analysis with ICU mortality as the dependent variable in patients with severe sepsis (*n *= 305). CI, confidence interval; COPD, chronic obstructive pulmonary disease; NA, not applicable; NYHA, New York Heart Association classification; OR, odds ratio; R, reference category; SAPS, Simplified Acute Physiology Score; SOFA, Sequential Organ Failure Assessment. ^a^Hosmer and Lemeshow χ^2 ^= 10.543 (*P *= 0.229). Nagelkerke pseudo-*R*^2 ^= 0.39.

## Discussion

The main finding of our study was that, although the overall prevalence of severe sepsis was lower in female patients admitted to the ICU than in male patients, female gender was independently associated with a higher risk of death in the ICU in patients with severe sepsis.

In our study, there were more male than female ICU admissions. This finding has been consistently reported in all the large epidemiologic studies in ICU patients [21-23]. The reason for this finding is unclear. One proposal has been that there may be a gender-related bias in the provision of care [5,8,24]. In a large multicenter Austrian cohort including 25,998 adult ICU patients, despite a higher severity of illness in women, men received an increased level of care and underwent more invasive procedures [5]. Another large single-center retrospective study, including 24,778 critically ill patients, reported that among patients 50 years or older, women were less likely than men to receive life-supporting treatments [8]. The differences in provision of care are probably not responsible alone for the higher ICU admission rates in men than women. Gender-related differences in immune response and in the presentation of critical illness cannot be excluded as an explanation for this finding [14,25].

The possible impact of gender on outcome from critical illness has been reported previously but with conflicting results [6,8,10]. In our study, gender had no impact on the ICU mortality rate in a large cohort of critically ill patients admitted to the region of Piedmont in Italy. To the best of our knowledge, our study is the first study to investigate the impact of gender in a representative sample of ICU patients admitted to a specific region. The absence of gender-related differences in outcome in our study does not preclude possible differences in outcome in specific subgroups of ICU patients. In agreement with our results, Valentin and colleagues found that outcome was similar in critically ill men and women admitted to 31 Austrian ICUs despite differences in the therapeutic approach according to gender [5]. However, Fowler and colleagues reported that female patients were more likely to die after critical illness [8] - although their study was limited by its single-center nature and retrospective design. Discrepancies between the results of these studies can also be explained, at least in part, by the differences in case mix.

In our study, the prevalence of severe sepsis was lower in women than in men. The design of our study does not enable us to elaborate on the possible pathophysiologic reasons for this finding. It has been reported that an increased estradiol level may enhance immune function in females [14,26,27]. Moreover, a predominance of anti-inflammatory mediators in women [14] may be responsible for a protective effect in female critically ill patients in terms of development of severe sepsis. Our results confirm those reported by Adrie and colleagues, in which women had a lower prevalence of severe sepsis [28]. Other results from Wichmann and colleagues showed a significantly lower incidence of severe sepsis/septic shock in female ICU patients between 60 and 79 years old compared with male patients [1]. In contrast, in a recent prospective multicenter cohort study of adult trauma patients with hemorrhagic shock, Sperry and colleagues noted that female gender was associated with a significant reduction in rates of multiple organ failure and nosocomial infection [12].

Differences in gender-related outcome may also exist in patients with severe sepsis. Schröder and colleagues reported higher survival rates in women with surgical sepsis than in men [14,25]. These authors also analyzed sex-related hormonal secretion and different patterns of proinflammatory and anti-inflammatory mediators in response to severe sepsis and found a more favorable hormonal and immunologic profile in women than in men. This study was limited, however, by the small number of patients and the inclusion of only surgical sepsis. In a large case-control study including 1,692 patients with severe sepsis, Adrie and colleagues found that mortality was higher in men than in women, especially in the subgroup of patients > 50 years old [7]. Several experimental studies have also reported a survival benefit in female septic animals compared with males [13,29]. Sexually dimorphic cytokine profiles, such as increased levels of proinflammatory cytokines, have been suggested to be responsible for this phenomenon [13,29]. Sex steroids can also modulate the inflammatory response and may subsequently influence outcome after septic challenge [30].

The results of our study are in contrast to some previous findings [7,14,25], with female gender being independently associated with a higher risk of death in patients with severe sepsis. Indeed, our data suggest a protective effect of female gender in terms of developing sepsis, with a lower prevalence of severe sepsis and septic shock. We suggest that females with an unfavorable immunologic profile are those who are more liable to develop severe sepsis and subsequently have a worse prognosis, but this hypothesis needs to be confirmed in large, prospective studies. Our results agree with those of Eachempati and colleagues, who demonstrated that female gender was an independent predictor of increased mortality in critically ill patients with documented infection [31]. More recently, Combes and colleagues analyzed the gender-related outcome of a mixed population of patients who developed nosocomial infections in the ICU and also reported that female gender was associated with an increased risk of ICU mortality [10].

Although this cohort of ICU patients is a representative sample from a specific region, our study has some limitations. First, the epidemiology of sepsis in this region may not be extrapolated to all ICUs in Italy. These results can also not be extrapolated to other parts of the world because genetic polymorphisms that could have an impact on mortality are not taken into account. Second, we considered only short-term outcome in terms of ICU mortality, and cannot therefore comment on the potential impact of gender on in-hospital mortality or longer term outcomes. Third, the relatively small sample size in the subgroup of patients with severe sepsis may be another limitation of our study. Finally, the multivariate analysis is limited to the variables considered for this analysis; however, we included a large number of variables relevant to outcome in this population, and adjusted for the center effect.

## Conclusions

In this large, regional Italian cohort of ICU patients, there were more male than female ICU admissions. The prevalence of severe sepsis was lower in women than in men, but female gender was independently associated with a higher risk of death in the ICU in patients with severe sepsis.

## Key messages

• In this cohort, the overall prevalence of severe sepsis was lower in female patients admitted to the ICU than in male patients.

• Female gender was independently associated with a higher risk of death in the ICU in patients with severe sepsis.

## Abbreviations

CI: confidence interval; OR: odds ratio; SAPS: Simplified Acute Physiology Score; SOFA: Sequential Organ Failure Assessment.

## Competing interests

The authors declare that they have no competing interests.

## Authors' contributions

VMR, LM, BB, SC, SL, and GF designed the study, contributed to recruitment of patients, and organized data collection during the study period. CF performed data-mining and organized the electronic data entry. CF and YS performed the statistical analysis. YS, CE, LM and VMR edited the manuscript. All authors read, revised, and accepted the submitted manuscript.

## Supplementary Material

Additional file 1 **a list of the contributing centers**. Click here for file

Additional file 2 **a table presenting the results of logistic regression analysis with ICU mortality as the dependent variable in the whole cohort**. Click here for file
