# Supplementary material for: The influence of gender on the epidemiology of and outcome from severe sepsis
Source: Crit Care. 2013 Mar 18;17(2):R50. doi: 10.1186/cc12570 (PMC3733421; doi:10.1186/cc12570)
Supplement: Additional file 1 — a list of the contributing centers. [file cc12570-S1.DOCX]

Appendix 1 List of the contributing centers

Ospedale S. Giovanni Battista-Molinette, Anestesia e Rianimazione 1-3, Torino (V.M.Ranieri, C.Bonetto, M.Lupo); Ospedale Civile, Chivasso (E. Castenetto, B.Scapino, L.Oberto); Ospedale Civile Ivrea (M. R. Salcuni, G.Belloni); Ospedale E. Agnelli, Pinerolo (L. Del Piano, M.Panzani, F.Berruto); Ospedale Civile S. Croce, Moncalieri (P. Buffa, G.Fiore, A.Cerutti); Ospedale Civile, Asti (S. Cardellino, S. Perno, E.Costanzo); Ospedale Civile, Ciriè (P. Giugiaro, S.Mulatero, M.Volpiano); Osp. S. Annunziata, Savigliano (G. Vai, E.Brizio); Ospedale degli Infermi, Rivoli (B. Barberis, B. Babuin); Ospedale S.Giovanni Antica Sede, Torino (L. Musso); Ospedale Martini, Torino (A. Parigi, M.Torta); Ospedale Maggiore della Carità, Novara (F. Della Corte, A. Gratarola); Ospedale S.Giovanni Battista-Molinette, Anestesia e Rianimazione 6, Torino (P.Donadio, M. Vaj, A.Santin, E.Cerutti); Osp. Molinette Neurorianimazione (M.Berardino); Osp. Molinette PSAR (P.Del Gaudio); IRCC Candiolo, Torino (F. De Bernardi, F.Bona); Ospedale San Giovanni Bosco, Torino (M.Maio, D.Silengo, D.Pietrobon, L.Rivalta); Centro Traumatologico Ortopedico (CTO), Torino (A.Miletto, D.Decaroli); Osp. Mauriziano Centrale Umberto I di Torino (V.Segola, M.Arobio, N.Lojacono, S.Minicucci); Ospedale Maggiore di Chieri (A.Mastroianni, G.Belforte); Ospedale Maria Vittoria di Torino (E.Manno); Osp. S.Croce e Carle di Cuneo (A.Locatelli, G.Cornara); Ospedale S. Luigi di Orbassano (TO) (G.Radeschi, F.Lodo, S.Furlan); Ospedale SS Antonio e Biagio di Alessandria (A.Pergolo, A.Cesareo, N.Vivaldi)
