# Supplementary material for: The influence of gender on the epidemiology of and outcome from severe sepsis
Source: Crit Care. 2013 Mar 18;17(2):R50. doi: 10.1186/cc12570 (PMC3733421; doi:10.1186/cc12570)
Supplement: Additional file 2 — a table presenting the results of logistic regression analysis with ICU mortality as the dependent variable in the whole cohort. [file cc12570-S2.DOC]

Additional file 2. Logistic regression analysis with ICU mortality as the dependent variable in the whole cohort.

|  | Univariate | | Multivariate* | |
| --- | --- | --- | --- | --- |
|  | OR (95% CI) | *P* value | OR (95% CI) | *P* value |
| Age (per year) | 1.03 (1.02-1.04) | < 0.001 | 1.01 (1.00-1.02) | 0.008 |
| Sex (Male) | 1.02 (0.86-1.2) | 0.840 | 1.07 (0.87-1.34) | 0.510 |
| Comorbidities |  |  |  |  |
| COPD | 0.84 (0.61-1.15) | 0.280 | 0.76 (0.50-1.16) | 0.280 |
| Renal failure (without dialysis) | 1.39 (1.04-1.85) | 0.025 | 0.77 (0.53-1.12) | 0.169 |
| Renal failure (with dialysis) | 2.91 (1.92-4.39) | < 0.001 | 1.67 (0.95-2.95) | 0.076 |
| Diabetes mellitus | 1.40 (1.14-1.72) | 0.001 | 1.13 (0.86-1.49) | 0.382 |
| Heart failure (NYHA III-IV) | 2.01 (1.56-2.59) | < 0.001 | 1.23 (0.88-1.74) | 0.224 |
| Metastatic cancer | 1.56 (1.12-2.16) | 0.008 | 1.21 (1.14-1.89) | <0.001 |
| Hematological cancer | 0.26 (0.16-0.44) | < 0.001 | 0.54 (0.29-1.03) | 0.061 |
| AIDS | 0.25 (0.06-0.99) | 0.050 | 0.62 (0.12-3.62) | 0.59 |
| SAPS II score (per point) | 1.08 (1.07-1.09) | < 0.001 | 1.06 (1.05-1.07) | <0.001 |
| Type of admission |  |  |  |  |
| Elective surgery | R | NA | R | NA |
| Emergency surgery | 4.73 (3.59-6.25) | < 0.001 | 1.42 (0.96-2.10) | 0.078 |
| Medical admission | 10.44 (8.10-13.46) | < 0.001 | 1.79 (1.14-2.80) | 0.011 |
| Referring facility |  |  |  |  |
| Other hospital | R | NA | R | NA |
| Other ICU | 1.25 (0.77-2.02) | 0.362 | 1.29 (0.69-2.39) | 0.429 |
| Emergency department | 1.42 (1.1-1.85) | 0.008 | 1.40 (1.01-1.94) | 0.039 |
| Surgical ward | 0.37 (0.29-0.49) | < 0.001 | 1.43 (0.97-2.11) | 0.072 |
| Medical ward | 1.41 (1.06-1.86) | 0.017 | 1.67 (1.16-2.41) | 0.006 |
| Reason for admission |  |  |  |  |
| Only monitoring | R | NA | R | NA |
| Intensive care | 10.17 (8.01-12.91) | < 0.001 | 2.44 (1.7-3.5) | <0.001 |
| Trauma | 0.65 (0.50-0.84) | 0.001 | 0.63 (0.44-0.89) | 0.009 |
| Sepsis syndromes during ICU stay |  |  |  |  |
| No sepsis | R | NA | R | NA |
| Sepsis | 1.19 (0.78-1.81) | 0.422 | 0.80 (0.44-1.44) | 0.456 |
| Severe sepsis | 3.82 (2.77-5.3) | < 0.001 | 1.70 (1.06-2.72) | 0.026 |
| Septic shock | 6.80 (4.83-9.57) | < 0.001 | 2.25 (1.49-3.49) | <0.001 |
| ICU-acquired sepsis | 4.75 (3.6-6.29) | < 0.001 | 1.18 (0.73-1.92) | 0.483 |

ICU=intensive care unit, CI= confidence interval, NYHA= New York Heart association classification, OR=odds ratio.

Hosmer and Lemeshow Chi square =11.9 (p=0.155). Nagelkerke pseudo R^2^=0.405
